# POPs in Polar Bears: Organochlorines Affect Bone Density

**DOI:** 10.1289/ehp.112-a1011

**Published:** 2004-12

**Authors:** David J. Tenenbaum

Both organochlorine chemicals—including a series of solvents and pesticides that have been banned in many parts of the world—and their metabolites have been linked to bone loss in a variety of species. Now, Christian Sonne of the Danish National Environmental Research Institute and colleagues have found a similar association in a new species **[*EHP* 112:1711–1716]**. Their work shows a link between organochlorine exposure and reduced bone mineral density among polar bears in East Greenland.

Organochlorines are among the chemicals known as persistent organic pollutants (POPs), which resist breakdown, store easily in fat, and bioaccumulate through the food chain. Organochlorines have been accumulating in the Arctic for decades, thanks to northward atmospheric transport. Due to the quirks of atmospheric transport, polar bears from East Greenland, Svalbard, and the Kara Sea have higher body burdens of organochlorines than polar bears from the rest of the Arctic.

In controlled studies of laboratory mammals, organochlorines including the pesticide DDT and the group of industrial chemicals known as polychlorinated biphenyls (PCBs) have caused changes in bone composition including reduced bone mineral density. Organochlorines have also been implicated in other bone diseases including periodontitis, a disorder of the gums and bones around the teeth.

The Danish researchers examined samples from 139 East Greenland polar bear skulls collected between 1892 and 2002. Samples collected in the period 1966–2002 were considered “post-pollution”—that is, they were collected after high concentrations of POPs began appearing in polar bear fat. Those collected in the period 1892–1932 were considered “pre-pollution.”

The researchers measured bone mineral density with dual X-ray absorptiometry—the same test used to detect osteoporosis in humans. They also examined organochlorine body burden in a subset of 58 samples collected between 1999 and 2002 for links with bone mineral density.

Among younger bears and adult males, bone mineral density was significantly reduced in post-pollution samples. The pattern was not seen among adult females, possibly due to an age-associated decline of estrogen, or because they were pregnant or nursing pups (both conditions mobilize bone calcium for the benefit of the offspring, and reduce bone density). Bone formation and resorption are governed by estrogen and androgen hormones, which help regulate both osteoblasts (cells that form bone cells) and osteoclasts (cells that break down bone cells).

In the 58 skulls collected between 1999 and 2002, exposure to total PCB compounds and to chlordane (a now-banned insecticide) both correlated with low bone mineral density among younger bears. In adult males, concentrations of dieldrin (another banned insecticide) and total DDT residues also correlated with low density. The researchers conclude that disruption of bone mineral composition correlates with the presence of PCBs, DDT residues, and other POPs among polar bears from East Greenland.

## Figures and Tables

**Figure:**
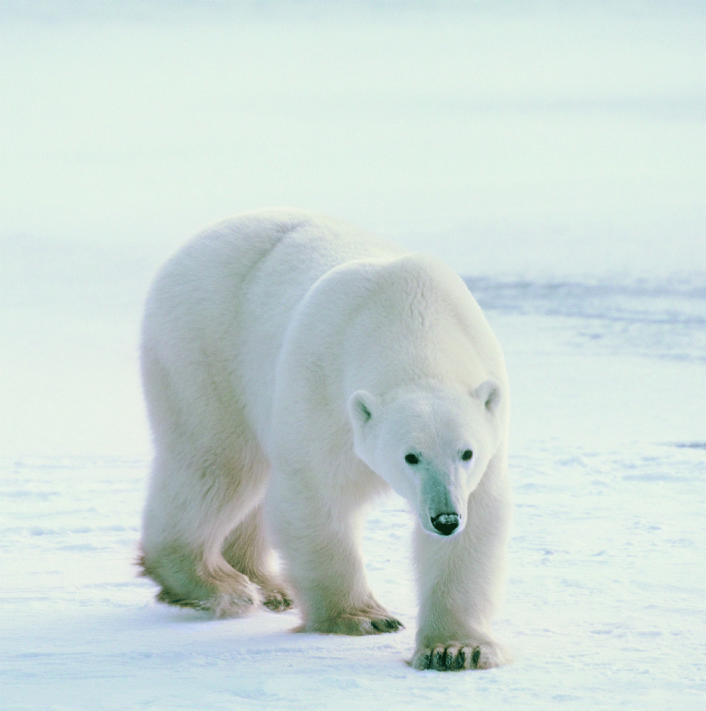
Bear-bones data. New research shows a link between Arctic organochlorine pollution and decreased bone mineral density in East Greenland polar bears.

